# Supplementary material for: Exosomal and Soluble Programed Death-Ligand 1 (PD-L1) Predicts Responses to Pembrolizumab in Patients with Extranodal NK/T-Cell Lymphoma
Source: Cancers (Basel). 2022 Nov 16;14(22):5618. doi: 10.3390/cancers14225618 (PMC9688922; doi:10.3390/cancers14225618)
Supplement: Supplementary file 1 [file cancers-14-05618-s001.zip › cancers-1986690-supplementary.pdf]

Supplementary Table S1. Antibodies for immunohistochemistry

Antibodies (clone)	Cat. No.	Clone	Company	Incubation time with primary antibody	Secondary and chromogen	Machine
Rabbit Anti-Human PD-L1/CD274 Monoclonal Antibody (Clone SP142)	M4424	SP142	Spring Bioscience	120min with Ventana BenchMark XT in 37 ℃	8min with OptiView Amplification Kit (860-099) + OptiView DAB IHC Detection kit (760-700)	BenchMark XT
Novocastra Liquid Mouse Monoclonal Antibody CD68	NCL-L-CD68	514H12	Leica	15min with Bond-max autoimmunostainer (Leica Biosystem, Melbourne, Australia)	10 min Bond-max autoimmunostainer (Leica Biosystem, Melbourne, Australia) using Bond™ Polymer refine detection, DS9800 (Vision Biosystems, Melbourne, Australia)	Bond max

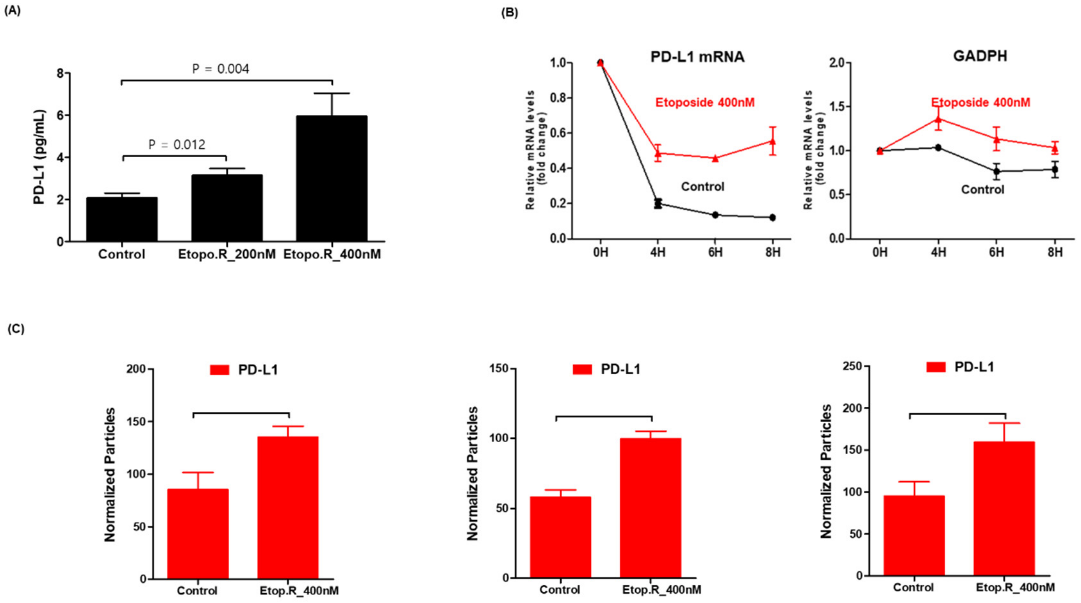

Supplementary Figure S1. Increased soluble and exosomal PD-L1 expression of SNK6R cells. (A) Increased levels of soluble PD-L1 in a culture media of SNK6R cells compared to control (parental SNK6 cells); (B) Increase of PD-L1 mRNA in SNK6R cells resistant to 400nM of etoposide compared to control (parental SNK6 cells); (C) Increased expression of PD-L1 in SNK6R cells-derived exosomes that were selected by exosomal markers, CD63, CD81 and CD9

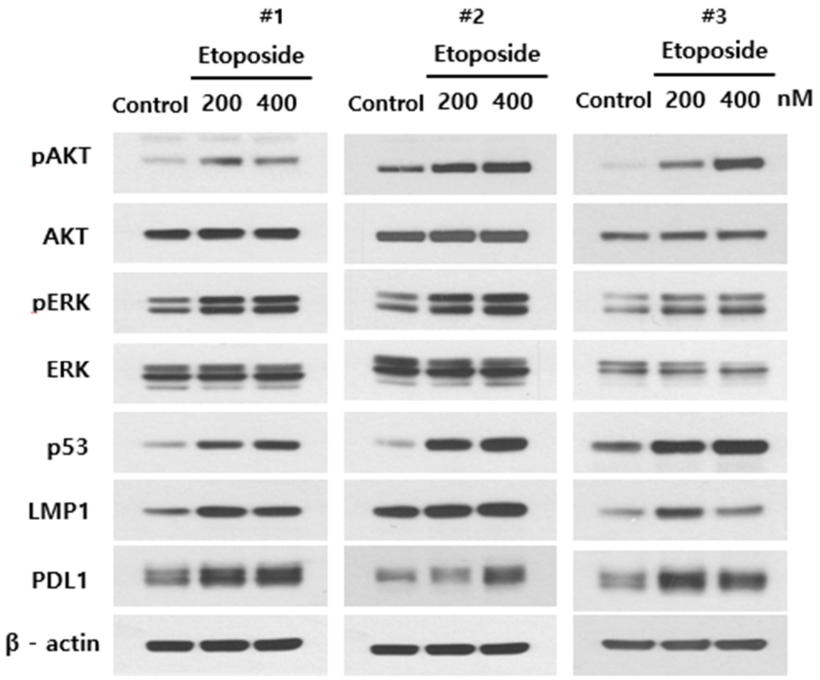

Supplementary Figure S2. Original blots of Figure 1.
